# C3-epi-25(OH)D3 percentage, not level, may be a potential biomarker to reflect its pathological increase in multiple diseases: a cross-sectional case–control study

**DOI:** 10.1038/s41598-023-50524-3

**Published:** 2023-12-27

**Authors:** Xiaohong Chen, Jie Tang, Dong Hu, Wenqiang Jiang, Jiafu Feng, Yuwei Yang

**Affiliations:** Department of Laboratory Medicine, School of Medicine, Mianyang Central Hospital, University of Electronic Science and Technology of China, Mianyang, 621000 People’s Republic of China

**Keywords:** Diseases, Medical research

## Abstract

National surveys in developed countries have examined the presence of C3-epimer of 25-hydroxyvitamin D3 [C3-epi-25(OH)D3]. However, controversy remains regarding its association with disease occurrence due to its high correlation with 25-hydroxyvitamin D3 [25(OH)D3]. This study aims to investigate whether %C3-epi-25(OH)D3 can serve as an indicator for this relationship with various diseases. A total of 3086 healthy participants and 4120 patients were included in this study. We investigated the association between C3-epi-25(OH)D3 and %C3-epi-25(OH)D3 levels with gender, age, and season; compared the performance of C3-epi-25(OH)D3 and %C3-epi-25(OH)D3 across different disease conditions; and explored the correlation between %C3-epi-25(OH)D3 and various diseases. Results indicated that C3-epi-25(OH)D3 varied significantly by gender, age, and season (*z*/*χ*2 = 3.765, 10.163, and 150.975, all *P* < 0.01), while only season for %C3-epi-25(OH)D3 (*χ*2 = 233.098, *P* < 0.001). In contrast to the significant decrease in C3-epi-25(OH)D3, %C3-epi-25(OH)D3 showed a significant increase in 8 out of 11 disease categories (*z* = 3.464 ~ 11.543, all *Padj* < 0.05). Similar opposite changes were also observed in most of the investigated 32 specific diseases. Moreover, an elevation in %C3-epi-25(OH)D3 was found to be significantly associated with 29 specific diseases both in univariate analysis (*OR* = 1.16 ~ 2.10, all *P* < 0.05) and after adjusting for gender, age, and season (*OR* = 1.15 ~ 1.50, all *P* < 0.05). However, after further adjustment for 25(OH)D3 levels, the association remained significant only for 15 specific diseases (*OR* = 1.11 ~ 1.50, all *P* < 0.05). Seasonal stratification analysis further supports the consistent association of %C3-epi-25(OH)D3 with disease across all or nearly all four seasons. In conclusion, %C3-epi-25(OH)D3 may better reflect the production of C3-epi-25(OH)D3 in disease conditions, thereby offering a more applicable approach to investigate its association with diseases. However, the interpretation of this relationship may be confounded by 25(OH)D3 as a potential covariate.

## Introduction

Vitamin D metabolites, lipid-soluble steroid derivatives, exert regulatory functions in calcium and phosphate metabolism^1^ as well as immune modulation^2^. Inadequate levels of vitamin D can result in reduced absorption of calcium and phosphorus, inflammation, oxidative stress, and impaired immune function. Consequently, this elevates the susceptibility to various diseases including osteomalacia, rickets, cardiovascular disorders, and autoimmune conditions^3,4^.

Over 50 metabolites are enzymatically synthesized in the metabolism of vitamin D^5^. Among these, 25-hydroxyvitamin D [25(OH)D] is recommended as the optimal marker for assessing in vivo vitamin D nutrition by authoritative bodies such as the Institute of Medicine (IOM)^6,7^, Endocrine Society^8^, and Japan Endocrine Society (JES)^9^. However, the presence of a metabolite form called C3-epimer of 25-hydroxyvitamin D [C3-epi-25(OH)D], which has negligible bioactivity, can lead to an overestimation of vitamin D storage^10^ and pose challenges in accurately evaluating vitamin D nutritional status^11^.

The formation of C3-epi-25(OH)D primarily occurs through 3β → 3α epimerization of 25(OH)D at the C3 position catalyzed by the enzyme 3-epimerase, leading to the inactivation of 25(OH)D^10^. This phenomenon of C3-epimerization was initially discovered in a glucuronic acid and cardenolidgenin conjugate in the 1960s^12^. However, it was not until 2004 that the specificity and properties of the C3-epi-25(OH)D3 metabolite were reported^13^, and its quantification could only be achieved through high-performance liquid chromatography-tandem mass spectrometry in 2006^14^. Prior to 2006, due to their similar spectroscopic characteristics and identical mass and fragmentation patterns, distinguishing between concentrations of both compounds using spectroscopy techniques was nearly impossible^15^. Given its very low levels in the general population, C3-epi-25(OH)D can easily be identified as signal noise when employing conventional mass spectrometry techniques unless its content is high.

To date, the determination of C3-epi-25(OH)D has been conducted for less than 15 years and has only been nationally surveyed in several developed countries^16,17^. Numerous studies have reported elevated levels of C3-epi-25(OH)D in various conditions and diseases, including infants, children, pregnant women, autoimmune disease, arthritis, diabetes mellitus, Alzheimer's disease, and thyroid disorders^18–20^. Due to its strong correlation with 25(OH)D levels over time^21^, most studies cautiously suggest that an increase in C3-epi-25(OH)D may result in an overestimation of vitamin D storage. However, the potential association between C3-epi-25(OH)D and diseases remains largely unknown. Our previous research was the first to demonstrate that C3-epi-25(OH)D could serve as a superior marker for predicting the severity of chronic kidney disease in rheumatoid arthritis patients^22^. Therefore, further investigation into this area is warranted.

## Methods

### Ethical approval and consent to participate.

This study adopted a cross-sectional case–control design and all methods were carried out in accordance with relevant guidelines and regulations or Declaration of Helsinki. It was performed in the Mianyang Central Hospital, affiliated to School of Medicine, University of Electronic Science and Technology of China. The Ethics Committee of Mianyang Central Hospital approved this study (approval No. S2018085). All participants or their parents signed informed consent.

### Participants

From November 2020 to June 2022, a total of 11,440 healthy adults and adult patients were included in the determination of 25(OH)D metabolites. Based on the inclusion and exclusion criteria outlined below, this study enrolled 3,086 healthy adults and 4,120 adult patients (2,302 males and 4,904 females), ranging in age from 22 to 90 years with an average age of 51.6 ± 15.0 years. The participant flow chart for this analysis is presented in Supplemental Fig. 1.

**Figure 1:**
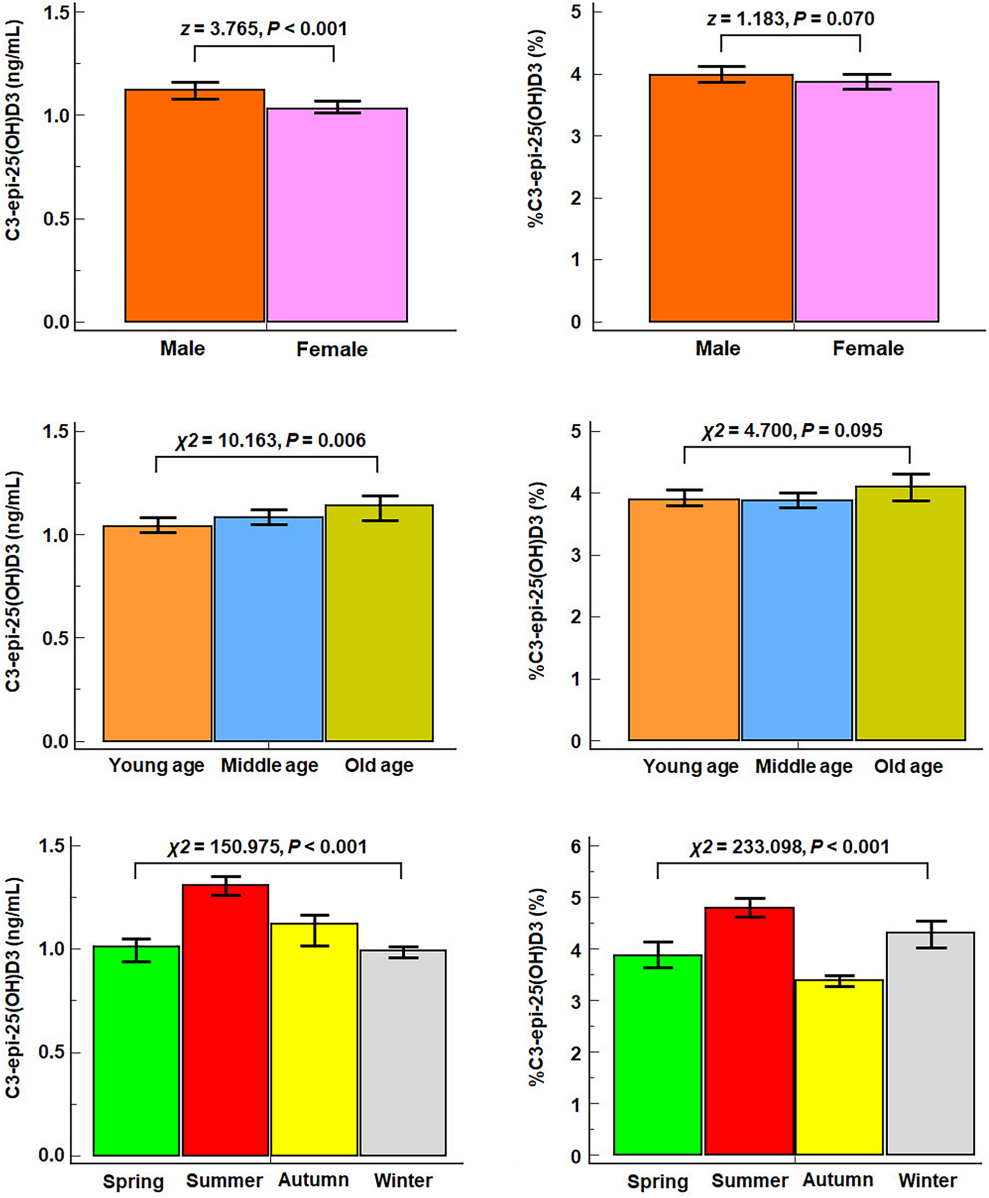
The gender, age, and season differences in C3-epi-25(OH)D3 level and percentage. Note: The Box height represents a median, and the top and bottom lines of I shape represent the 95%CI of the median. By the Mann–Whitney U test for two gender groups and the Kruskal–Wallis for multiple age and seasonal groups test, our results showed that C3-epi-25(OH)D3 had significant differences in gender, age, and season. But %C3-epi-25(OH)D3 had only seasonal differences.

(1) The inclusion and exclusion criteria of healthy controls.

The inclusion criteria for healthy adults were as follows: (1) Above 18 years old; (2) Attendance at the Health Management Center for a general medical examination; (3) Absence of visible physical impairment or disability; (4) Normal cardiac, pulmonary, hepatic, and renal function as well as no abnormalities in routine blood and urine examinations; (5) Total serum levels of 25(OH)D more than 20 ng/mL.

The exclusion criteria for healthy adults were as follows: (1) Diagnosis of any definite disease or illness after general medical examination; (2) Presence of obvious abnormalities in hepatic and renal function tests, complete blood count, and routine urine test results; (3) Presence of obvious abnormalities on ultrasound or imaging examinations (if performed); (4) Ostensible healthy adults with total 25(OH)D level less than 20 ng/mL; and (5) Taking any medication or VitD supplementation within 1 month.

(2) The inclusion and exclusion criteria of various diseases.

The inclusion criteria for adult patients were as follows: (1) Above 18 years old; (2) Attendance at specialist clinics or hospital admission; (3) Diagnosis of disease strictly adhered to the cluster coding of the WHO International Classification of Diseases^23^; and (4) Single disease diagnosis whenever possible, without comorbidities or severe complications.

The exclusion criteria for adult patients were as follows: (1) Presence of more than two comorbidities; (2) Severe complications in other tissues or organs; (3) Long-term use of gastrointestinal drugs (containing sucralfate or aluminum hydroxide), glucocorticoids within the past week, or vitamin D supplementation within the past month.

### Sample collecting and processing

Aliquots of 3.0 ml fasting venous blood were collected in an SST-II vacuum tube (BD, USA) in the early morning and centrifuged at about 1500 × g for 10 min to separate serum for determining levels of 25(OH)D2, 25(OH)D3, and C3-epi-25(OH)D3. If not immediately determined, store the serum sample at 2 ~ 8℃ for less than seven days^24^.

The lipid-soluble vitamin assay kits (Cat.No.:1001040, FINDBiotech, Chengdu FIND Medical laboratory Co., LTD, CHN) were used for sample pretreatment to obtain a supernatant for chromatographic analysis, as well as to provid the standards. On the day of measurement, 200 μl serum sample was mixed with 10 μl internal standard (a mixture solution of 25[OH]D3-d6 and C3-epi-25(OH)D3-[2H3]), vortexed with 1.0 ml tertbutyl methyl ether release agent (CNW, Germany) for 5 min, centrifuged at 12,000 × g for 5 min. The supernatant (800 μl) was dried using an MD200-1A Nitrogen Evaporator (Allsheng, China), and the resulting substance was redissolved in 100 μl methanol solution containing 0.1% formic acid. After vortex mixing for 2 min and centrifugation at 12,000 × g for 5 min, a sample of the supernatant (50 μl) was collected to measure levels of 25(OH)D metabolites.

### Isolation, identification and measurement of 25(OH)D3 metabolites

The supernatants were used to measure 25(OH)D3 metabolite levels on a Jasper™ HPLC–MS/MS (Shimadzu, Japan)/AB SCIEX™ 4500MD triple quadrupole mass spectrometer (ABI, USA). As an internal standard method, the peak area ratio of mass spectrum between the sample and the internal standard was calculated. Specifically, 25[OH] D3-D6 and C3-epi-25(OH)D3-[2H3] were used as internal standard for 25(OH)D3 and C3-epi-25(OH)D3, respectively. The peak area ratio of five gradient concentration standards (containing 5 ng/ml, 12.5 ng/ml, 25 ng/ml, 50 ng/ml, and 100 ng/ml of 25(OH)D3, respectively) was utilized for the generation of a standard curve, which was utilized for the concentration quantification of the 25(OH)D3 and C3-epi-25(OH)D3. Based on measured results, %C3-epi-25(OH)D3 was defined as the C3-epi-25(OH)D3 concentration divided by the sum of the C3-epi-25(OH)D3 and 25(OH)D3 concentrations.

HPLC conditions: F5 column, 40℃ temperature. Mobile phase A of 0.1% formic acid aqueous solution and mobile phase B of methanol solution containing 0.1% formic acid were used for density gradient elution at a flow rate of 0.6 ml/min. The details of density gradient elution: Mobile phase A and B of 35% and 65% during 0 to 1 min, 10% and 90% during 1 to 1.5 min, 5% and 95% during 1.5 to 4.0 min, 35% and 65% during 4.0 to 5.0 min.

MS conditions: curtain gas 25psi, atomized gas 40psi, heated gas 30psi, collision gas 6psi, solvent-removal temperature 350℃, impact chamber injection voltage 6 V, declustering potential 130 V, collision voltage 16 V. The analysis mode utilized positive ion multiple reaction monitoring with APCI ion source and a 40 ms residence time. Chromatograms and monitoring data were analyzed using Analyst® MD software v1.6.3 and Multiquant™ MD software v3.0.2.

This method has been previously reported^22^. C3-epi-25(OH)D3, a C3-epimeric metabolite of 25(OH)D3, was identified via monitoring the Q1/Q3 mass = 383:3 Da/365:3 Da ion pairs, with the retention time of 3.48 min, which different from that of 25(OH)D3 at 3.40 min (as shown in Supplemental Fig. 2). After a rigorous performance verification, the lower limit of quantitation for 25(OH)D3 and C3-epi-25(OH)D3 were determined to be 1.00 ng/ml and 0.40 ng/ml, respectively.

**Figure 2:**
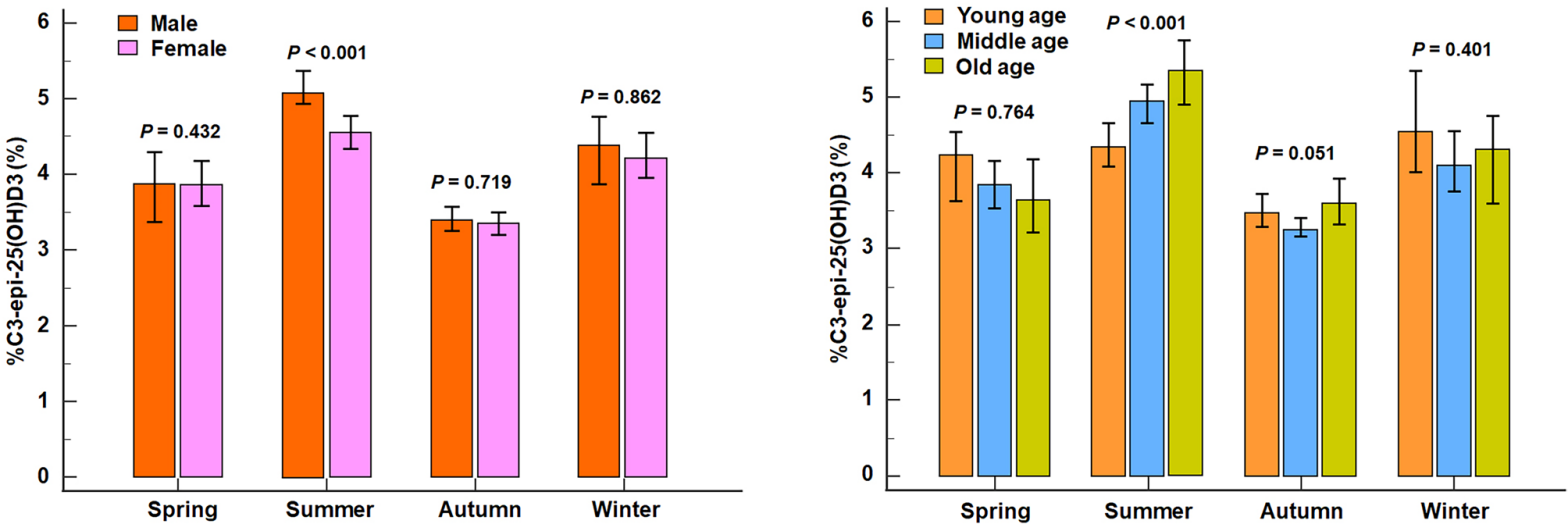
Seasonally stratified verification of %C3-epi-25(OH)D3 differences in gender and age. Note: The Box height represents a median, and the top and bottom lines of I shape represent the 95%CI of the median. By the Mann–Whitney U test for two gender groups and the Kruskal–Wallis test for multiple age groups, our results showed that %C3-epi-25(OH)D3 had no differences in gender and age during the other seasons except for in summer.

### Statistical analysis

Statistical analysis was conducted using MedCalc software v20.1 (MedCalc, Belgium) and SPSS software v22.0 (SPSS, USA). Non-normal distribution was confirmed by the D'agostino-Pearson test and measurement data were presented as *median* (*P*_*25*_, *P*_*75*_) [*min*, *max*]. In order to carry out the subsequent analysis favourably, we used the minimum replacement method to fill in the measurement results below the quantitative limit. Gender differences were analyzed using Mann–Whitney U test. Age and seasonal differences among multiple groups were analyzed using Kruskal–Wallis test. Pairwise comparison was performed by the Post-Hoc multiple comparisons, and the adjusted P value (*Padj*) was employed to determine the significance of the difference. The relationship between %C3-epi-25(OH)D3 and multiple diseases was assessed using multinomial logistic regression, with odds ratios (ORs) and 95% confidence intervals (CIs) used to determine the degree of association. A *P* or *Padj* value below 0.05 was considered significant.

## Results

### Influence of gender, age, and season on C3-epi-25(OH)D3 physiological generation

We investigated the impact of gender, age, and season on the physiological generation of C3-epi-25(OH)D3 in a sample size of 3086 healthy adult controls. The group settings were as follows: male (n = 1476) and female (n = 1610); young age (n = 935, 18 to 44 years old), middle age (n = 1629, 45 to 64 years old) and old age (n = 522, above 65 years old); spring (n = 617), summer (n = 905), autumn (n = 880) and winter (n = 684). Results (Fig. 1) showed that the gender, age, and season differences were also observed in C3-epi-25(OH)D3 levels (*z*/*χ*2 = 3.765, 10.163 and 150.975, all *P* < 0.01). However, %C3-epi-25(OH)D3 only exhibited a season difference (*χ*2 = 233.098, *P* < 0.001), with no significant gender difference (*z* = 1.183, *P* = 0.070), and age difference (*χ*2 = 4.700, *P* = 0.095). Furthermore, we found no age or gender differences in %C3-epi-25(OH)D3 levels during most seasons, except for summer (age: *χ2* = 19.858, *P* < 0.001; gender: *z* = 4.064, all *P* < 0.001) (shown in Fig. 2). These results suggest that while C3-epi-25(OH)D3 levels are affected by various factors, %C3-epi-25(OH)D3 may be a useful marker for evaluating its generation in most situations.

### Differential performance of C3-epi-25(OH)D3 levels and percentages in various disease categories

After observing that there were no significant differences in %C3-epi-25(OH)D3 levels based on gender or age, and no notable seasonal distribution variations between disease and healthy cases (*χ*2 = 45.165, *P* = 0.077), we proceeded to compare the overall %C3-epi-25(OH)D3 levels between the disease category and healthy controls. Overall, C3-epi-25(OH)D3 was detectable in 86.3% of the healthy adult controls, and in 83.2% of the patients.

Compared to the healthy controls (as shown in Table 1): 25(OH)D3 levels decreased in all the 11 disease categories (*z* = −10.155 ~ −39.989, all *Padj* < 0.001); C3-epi-25(OH)D3 levels significantly decreased in 10 disease categories (*z* = −3.530 ~ −16.443, all *Padj* < 0.05); however, %C3-epi-25(OH)D3 significantly increased in 8 disease categories (*z* = 3.464 ~ 11.543, all *Padj* < 0.05). These findings suggest that the decline observed in C3-epi-25(OH)D3 levels within these disease states is likely attributed to its strong correlation with 25(OH)D3 levels; whereas %C3-epi-25(OH)D3 can effectively demonstrate the percentage increase in C3-epi-25(OH)D3 generation.

**Table 1:** C3-epi-25(OH)D3 measured level and its proportion in total 25(OH)D3 metabolites {Upper row: *Median*; Middle row: (*P*_25_, *P*_75_); lower row: [*min*, *max*]}.

Factor	n	25(OH)D3 (ng/ml)	C3-epi-25(OH)D3 (ng/ml)	%C3-epi-25(OH)D3 (%)
Healthy controls	3086	25.9 (22.7, 30.5) [20.0, 58.0]	1.07 (0.74, 1.50) [< 0.40, 4.05]	3.89 (2.70, 5.37) [0.07, 8.25]
Obstetric diseases	214	17.0* (12.8, 22.9) [6.2, 43.3]	0.87* (0.62, 1.35) [< 0.40, 6.01]	5.02* (3.41, 6.75) [0.71, 23.02]
Gynecological diseases	287	16.9* (13.1, 23.4) [5.9, 49.8]	0.80* (0.53, 1.15) [< 0.40, 3.32]	4.31* (3.05, 5.80) [0.60, 21.70]
Skeletal diseases	1782	16.7* (11.8, 22.5) [2.2, 52.0]	0.78* (0.51, 1.21) [< 0.40, 7.92]	4.60* (3.21, 6.34) [0.13, 27.86]
Respiratory diseases	95	16.1* (11.1, 21.9) [4.3, 44.1]	0.85* (0.53, 1.27) [< 0.40, 2.71]	5.06* (3.57, 7.57) [0.36, 17.02]
Muscular diseases	317	15.5* (12.2, 20.9) [3.3, 51.8]	0.71* (0.46, 1.12) [< 0.40, 3.22]	4.39* (2.93, 6.23) [0.77, 15.38]
Endocrine diseases	703	15.9* (11.6, 21.5) [2.2, 52.5]	0.75* (0.46, 1.18) [< 0.40, 4.59]	4.61* (2.97, 6.37) [0.89, 21.70]
Skin diseases	59	14.3* (10.7, 20.6) [6.0, 32.2]	0.78* (0.42, 1.19) [< 0.40, 3.49]	5.02 (2.73, 7.59) [0.83, 26.56]
Neurological disorders	111	14.7* (10.6, 19.7) [4.2, 37.3]	0.73* (0.47, 1.01) [< 0.40, 3.14]	4.73 (2.99, 6.20) [0.65, 15.38]
Digestive diseases	66	15.4* (11.0, 20.9) [5.9, 34.6]	0.82 (0.57, 1.18) [0.13, 5.66]	4.94 (3.67, 6.81) [1.23, 16.01]
Cardiovascular disease	146	15.7* (12.2, 22.4) [3.9, 56.0]	0.77* (0.48, 1.10) [< 0.40, 3.51]	4.72* (3.14, 6.01) [0.80, 25.30]
Autoimmune diseases	340	15.7* (11.0, 20.5) [2.2, 46.9]	0.76* (0.45, 1.23) [< 0.40, 4.20]	4.73* (3.10, 6.34) [0.36, 18.00]
*χ*^*2*^, *P*		2649.543, < 0.001	502.776, < 0.001	206.887, < 0.001

*Padj < 0.05. The Kruskal–Wallis test and Post-Hoc multiple comparisons was used for multiple groups comparison and pairwise comparison, respectively. The results suggest that the decrease of C3-epi-25(OH)D3 levels in disease states showed a strong correspondence with the decrease of 25(OH)D3 levels, while the relative increase in %C3-epi-25(OH)D3 may be effectively demonstrate the percentage increase in C3-epi-25(OH)D3 generation.

Moreover, there were no significant differences observed in the levels of 25(OH)D3 levels (|*z*|= 0.023 ~ 2.863, all *Padj* > 0.05), C3-epi-25(OH)D3 levels (|*z*|= 0.004 ~ 3.305, all *Padj* > 0.05), and %C3-epi-25(OH)D3 (|*z*|= 0.088 ~ 3.304, all *Padj* > 0.05) among the various disease categories.

### Differential performance of C3-epi-25(OH)D3 levels and percentages in various diseases

**Figure 3:**
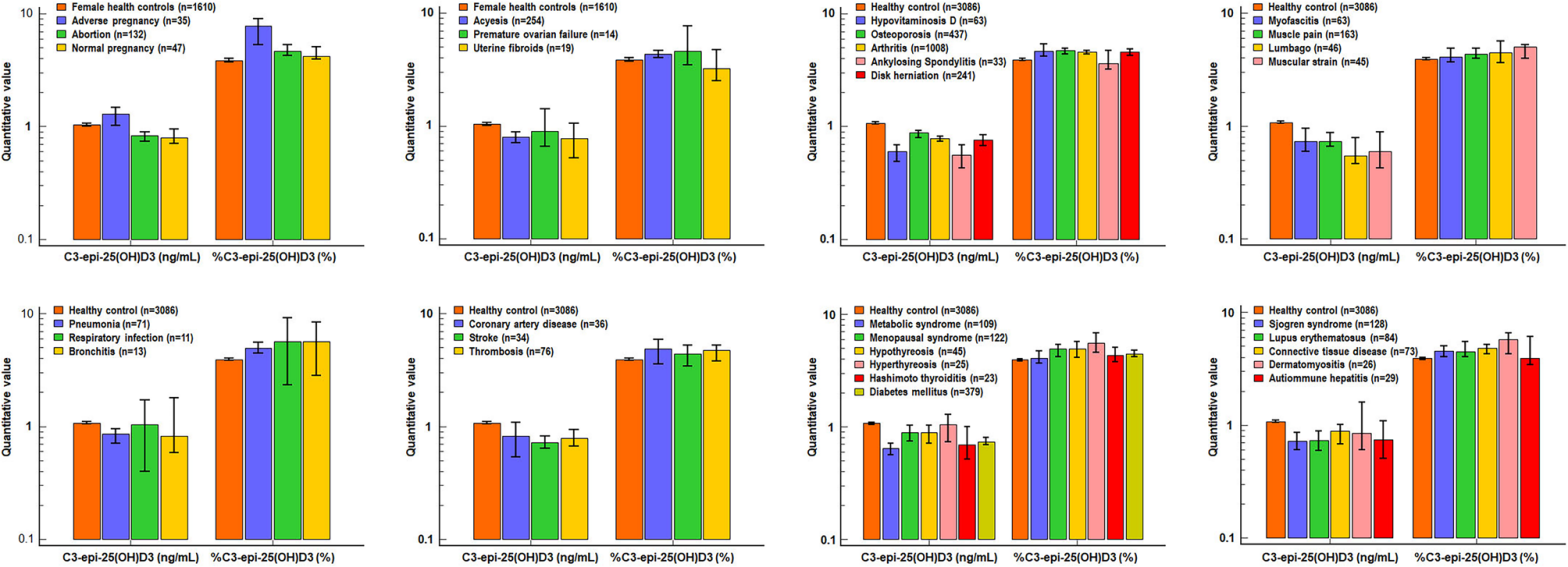
The Box plot of C3-epi-25(OH)D3 level and percentage in various diseases. Note: The Box height represents a median, and the top and bottom lines of I shape represent the 95%CI of the median. The Kruskal–Wallis test and Post-Hoc multiple comparisons was used for multiple groups comparison and pairwise comparison, respectively. In most diseases, the overall level of C3-epi-25(OH)D3 levels exhibited a decreasing trend, possibly due to its highly-tracking to 25(OH)D3 levels. However, %C3-epi-25(OH)D3 showed an increasing trend, suggesting that it may accurately reflect the pathological increase in C3-epi-25(OH)D3 generation.

We conducted a crude analysis of the changes in %C3-epi-25(OH)D3 and C3-epi-25(OH)D3 levels across 32 specific diseases, which are attached to the 8 disease categories that exhibited significant changes. In most specific diseases (Fig. 3), we observed similar opposite trends in %C3-epi-25(OH)D3 and C3-epi-25(OH)D3 levels as seen in the disease categories, except for adverse pregnancy patients where both C3-epi-25(OH)D3 levels (*z* = 1.959, *Padj* = 0.301) and %C3-epi-25(OH)D3 (*z* = 6.275, *Padj* < 0.001) increased. Furthermore, there were diverse patterns of %C3-epi-25(OH)D3 and C3-epi-25(OH)D3 levels among 26 specific diseases (as shown in Table 2): 3 (11.5%) of diseases exhibited significant differences only in %C3-epi-25(OH)D3, 11 (42.3%) exhibited significant differences only in C3-epi-25(OH)D3 levels, and 12 (46.2%) exhibited significant differences in both. The inconsistent changes in %C3-epi-25(OH)D3 and C3-epi-25(OH)D3 levels across various disease conditions suggest that %C3-epi-25(OH)D3 may serve as a more appropriate indicator for investigating the association between C3-epi-25(OH)D3 and diseases.

**Table 2:** Post-HOC results for C3-epi-25(OH)D3 levels and percentages between disease and health conditions (*z*, *Padj*), and the consistency on their difference significance.

Diseases	C3-epi-25(OH)D3*	%C3-epi-25(OH)D3*	Variation trend	Whether both P < 0.05
Adverse pregnancy	**1.959, 0.301**	**6.275, < 0.001**	**Both increased**	**No**
Abortion	−5.160, < 0.001	4.159, < 0.001	Inversely changed	Yes
Normal pregnancy	−**3.190, 0.009**	**2.262, 0.142**	**Inversely changed**	**No**
Acyesis	−7.408, < 0.001	3.696, 0.001	Inversely changed	Yes
Hypovitaminosis D	−7.715, < 0.001	3.134, 0.026	Inversely changed	Yes
Arthritis	−14.325, < 0.001	8.884, < 0.001	Inversely changed	Yes
Ankylosing Spondylitis	−**5.043, < 0.001**	**0.013, 1.000**	**Inversely changed**	**No**
Disk herniation	−7.946, < 0.001	5.206, < 0.001	Inversely changed	Yes
Osteoporosis	−7.255, < 0.001	6.918, < 0.001	Inversely changed	Yes
Muscle pain	−6.829, < 0.001	3.611, < 0.001	Inversely changed	Yes
Myofascitis	−**4.379, < 0.001**	**1.751, 0.799**	**Inversely changed**	**No**
Muscular strain	−**4.948, < 0.001**	**1.716, 0.861**	**Inversely changed**	**No**
Lumbago	−**5.505, < 0.001**	**1.544, 1.000**	**Inversely changed**	**No**
Pneumonia	−3.712, < 0.001	4.394, < 0.001	Inversely changed	Yes
Coronary artery disease	−**2.702, 0.041**	**2.256, 0.144**	**Inversely changed**	**No**
Stroke	−**4.167, < 0.001**	**1.807, 0.425**	**Inversely changed**	**No**
Thrombosis	−**1.498, < 0.001**	**2.410, 0.096**	**Inversely changed**	**No**
Metabolic syndrome	−**7.748, < 0.001**	**1.633, 1.000**	**Inversely changed**	**No**
Menopausal syndrome	−4.061, 0.001	3.212, 0.028	Inversely changed	Yes
Hashimoto thyroiditis	−**3.291, 0.021**	**1.439, 1.000**	**Inversely changed**	**No**
Hyperthyreosis	−**0.903, 1.000**	**3.215, 0.027**	**Inversely changed**	**No**
Diabetes mellitus	−11.079, < 0.001	5.593, < 0.001	Inversely changed	Yes
Sjogren syndrome	−7.309, < 0.001	3.171, 0.023	Inversely changed	Yes
Dermatomyositis	−**1.340, 1.000**	**3.787, 0.002**	**Inversely changed**	**No**
Lupus erythematosus	−5.245, < 0.001	3.384, 0.011	Inversely changed	Yes
Connective tissue disease	−**3.783, 0.002**	**2.788, 0.080**	**Inversely changed**	**No**

*The statistic z and significance *P* value of the Post-Hoc multiple comparisons in Kruskal–Wallis test. Only the specific diseases with significant differences in C3-epi-25(OH)D3 level and/or percentage compared with healthy controls are listed here. C3-epi-25(OH)D3 is decreased with 25(OH)D3 reduction in almost all diseases (except adverse pregnancy), but the generation rate of C3-epi-25(OH)D3 is increased in any disease condition. Among the 26 specific diseases, only 12(46.2%) had significant differences compared with health controls in both C3-epi-25(OH)D3 levels and percentages. Significant values are in bold.

### Crude correlation analysis between %C3-epi-25(OH)D3 and various specific diseases

The odds ratio (OR) of %C3-epi-25(OH)D3 for various diseases was analyzed using multinomial logistic regression, with health controls as a reference. Subsequently, additional adjustments were made for gender, age, season, and 25(OH)D3 level. Among the 32 diseases investigated (as presented in Table 3), a significant association was observed between an increase in %C3-epi-25(OH)D3 and 29 diseases, both before and after adjusting for gender, age, and season (before: *OR* = 1.16 ~ 2.10, all *P* < 0.05; after: *OR* = 1.15 ~ 1.50, all *P* < 0.05). However, upon further adjustment for 25(OH)D3 levels, the association remained statistically significant only for 15 specific diseases (*OR* = 1.11 ~ 1.50, all *P* < 0.05). These findings suggest that an elevated %C3-epi-25(OH)D3 level may be linked to disease occurrence irrespective of gender, age, and season; however, caution should be exercised when considering adjustments for 25(OH)D3 levels as it attenuates the relationship between %C3-epi-25(OH)D3 and disease.

**Table 3:** Odds ratios of %C3-epi-25(OH)D3 in multiple diseases.

Disease	Crude analysis			Adjusted analysis (1) *			Adjusted analysis (2) *		
	OR (95%CI)	Wald χ2	P	OR (95%CI)	Wald χ2	P	OR (95%CI)	Wald χ2	P
Adverse pregnancy	**2.10(1.40, 3.19)**	**12.558**	** < 0.001**	**1.50(1.35, 1.66)**	**59.439**	** < 0.001**	**1.50(1.28, 1.77)**	**24.321**	** < 0.001**
Abortion	**1.66(1.26, 2.19)**	**12.787**	** < 0.001**	**1.31(1.22, 1.41)**	**53.074**	** < 0.001**	**1.28(1.14, 1.44)**	**17.953**	** < 0.001**
Normal pregnancy	**1.21(1.05, 1.40)**	**6.588**	**0.010**	**1.21(1.06, 1.37)**	**8.529**	**0.003**	1.15(0.98, 1.35)	2.943	0.086
Acyesis	**1.42(1.19, 1.70)**	**15.255**	** < 0.001**	**1.21(1.13, 1.29)**	**33.957**	** < 0.001**	**1.38(1.24, 1.69)**	**9.800**	**0.002**
Premature ovarian failure	**1.17(1.10, 1.26)**	**22.006**	** < 0.001**	**1.43(1.22, 1.68)**	**19.976**	** < 0.001**	1.11(1.00, 1.22)	3.917	0.050
Uterine fibroids	0.98(0.76, 1.25)	0.040	0.841	0.98(0.77, 1.25)	0.022	0.883	0.90(0.68, 1.18)	0.582	0.445
Hypovitaminosis D	**1.23(1.11, 1.36)**	**16.042**	** < 0.001**	**1.24(1.12, 1.37)**	**17.774**	** < 0.001**	1.08(0.97, 1.20)	1.820	0.177
Osteoporosis	**1.24(1.19, 1.29)**	**95.943**	** < 0.001**	**1.23(1.17, 1.28)**	**81.746**	** < 0.001**	**1.17(1.11, 1.23)**	**33.192**	** < 0.001**
Arthritis	**1.22(1.18, 1.26)**	**138.725**	** < 0.001**	**1.20(1.16, 1.24)**	**109.507**	** < 0.001**	**1.11(1.07, 1.17)**	**22.783**	** < 0.001**
Ankylosing Spondylitis	1.04(0.87, 1.23)	0.187	0.665	1.06(0.90, 1.26)	0.521	0.470	0.95(0.79, 1.15)	0.256	0.613
Disk herniation	**1.21(1.14, 1.28)**	**43.108**	** < 0.001**	**1.18(1.11, 1.25)**	**31.626**	** < 0.001**	**1.10(1.03, 1.17)**	**8.285**	**0.004**
Myofascitis	**1.25(1.11, 1.41)**	**14.027**	** < 0.001**	**1.22(1.09, 1.35)**	**12.745**	** < 0.001**	1.13(0.99, 1.28)	3.456	0.063
Muscle pain	**1.24(1.15, 1.34)**	**30.049**	** < 0.001**	**1.18(1.11, 1.27)**	**23.235**	** < 0.001**	1.08(0.98, 1.19)	2.627	0.105
Lumbago	**1.17(1.01, 1.35)**	**4.290**	**0.038**	**1.15(1.01, 1.31)**	**4.311**	**0.038**	1.01(0.86, 1.18)	0.010	0.919
Muscular strain	**1.16(1.01, 1.35)**	**4.132**	**0.042**	**1.15(1.01, 1.32)**	**4.359**	**0.037**	1.03(0.89, 1.20)	0.187	0.666
Pneumonia	**1.51(1.21, 1.88)**	**13.159**	** < 0.001**	**1.30(1.20, 1.41)**	**43.123**	** < 0.001**	**1.42(1.10, 1.83)**	**7.122**	**0.008**
Respiratory infection	**1.44(1.29, 1.61)**	**43.956**	** < 0.001**	**1.41(1.23, 1.61)**	**25.191**	** < 0.001**	1.13(0.97, 1.31)	2.366	0.124
Bronchitis	**1.70(1.39, 2.09)**	**25.815**	** < 0.001**	**1.35(1.15, 1.57)**	**14.284**	** < 0.001**	1.32(0.98, 1.73)	3.075	0.079
Coronary artery disease	**1.33(1.17, 1.52)**	**19.130**	** < 0.001**	**1.27(1.14, 1.42)**	**18.373**	** < 0.001**	1.16(0.97, 1.38)	2.759	0.097
Stroke	**1.28(1.17, 1.48)**	**12.276**	** < 0.001**	**1.20(1.05, 1.37)**	**7.124**	**0.008**	1.03(0.85, 1.24)	0.066	0.797
Thrombosis	**1.20(1.08, 1.34)**	**10.650**	**0.001**	**1.25(1.13, 1.38)**	**18.538**	** < 0.001**	**1.23(1.05, 1.44)**	**6.743**	**0.009**
Metabolic syndrome	**1.18(1.08, 1.29)**	**13.782**	** < 0.001**	**1.22(1.12, 1.33)**	**22.506**	** < 0.001**	**1.12(1.01, 1.24)**	**4.305**	**0.038**
Menopausal syndrome	**1.21(1.11, 1.31)**	**20.357**	** < 0.001**	**1.21(1.12, 1.30)**	**22.524**	** < 0.001**	**1.14(1.03, 1.27)**	**6.592**	**0.010**
Hypothyreosis	**1.19(1.04, 1.36)**	**6.547**	**0.011**	**1.20(1.06, 1.35)**	**8.571**	**0.003**	1.13(0.98, 1.30)	2.889	0.089
Hyperthyroidism	**1.34(1.16, 1.55)**	**16.033**	** < 0.001**	**1.33(1.17, 1.52)**	**18.842**	** < 0.001**	**1.27(1.08, 1.49)**	**8.297**	**0.004**
Hashimoto thyroiditis	1.12(0.92, 1.36)	1.204	0.272	1.15(0.95, 1.39)	2.045	0.153	1.04(0.85, 1.29)	0.162	0.687
Diabetes mellitus	**1.26(1.20, 1.32)**	**86.669**	** < 0.001**	**1.22(1.16, 1.27)**	**67.408**	** < 0.001**	**1.11(1.03, 1.19)**	**7.746**	**0.005**
Sjogren syndrome	**1.21(1.11, 1.31)**	**20.454**	** < 0.001**	**1.19(1.10, 1.29)**	**19.566**	** < 0.001**	1.08(0.98, 1.20)	2.330	0.127
Lupus erythematosus	**1.37(1.25, 1.50)**	**48.026**	** < 0.001**	**1.35(1.26, 1.45)**	**65.990**	** < 0.001**	**1.23(1.11, 1.37)**	**14.299**	** < 0.001**
Connective tissue disease	**1.23(1.11, 1.37)**	**15.057**	** < 0.001**	**1.21(1.10, 1.33)**	**14.579**	** < 0.001**	1.14(0.99, 1.30)	3.287	0.070
Dermatomyositis	**1.50(1.31, 1.71)**	**35.535**	** < 0.001**	**1.38(1.25, 1.53)**	**37.816**	** < 0.001**	**1.30(1.13, 1.50)**	**12.796**	** < 0.001**
Autiommune hepatitis	**1.36(1.18, 1.57)**	**17.257**	** < 0.001**	**1.30(1.14, 1.47)**	**16.706**	** < 0.001**	**1.20(1.03, 1.39)**	**5.566**	**0.018**

The multinomial logistic regression was used. * (1) Adjusted by gender, age, season; (2) Adjusted by gender, age, season and 25(OH)D3 level. An increase in %C3-epi-25(OH)D3 can predict an increased risk of 29/32 (90.6%) specific diseases, regardless of adjusted for gender, age, and season. But adjustment for 25(OH)D3 level significantly reduced the ability of %C3-epi-25(OH)D3 to predict disease risk. Significant values are in bold.

### Seasonally stratified verification of the correlation between %C3-epi-25(OH)D3 and various specific diseases

To investigate the seasonal variation in the association between %C3-epi-25(OH)D3 and disease, we selected 10 specific diseases with over 100 cases from this survey. As depicted in Table 4, a consistent association was observed between %C3-epi-25(OH)D3 levels and osteoporosis as well as arthritis across all four seasons. Moreover, significant associations were also found between %C3-epi-25(OH)D3 and eight other diseases (abortion, acyesis, disk herniation, muscle pain, metabolic syndrome, menopausal syndrome, diabetes) in most seasons. These findings suggest that an elevated level of %C3-epi-25(OH)D3 is consistently associated with disease occurrence regardless of seasonal influences.

**Table 4:** Odds ratio of %C3-epi-25(OH)D3 to 10 specific diseases in different seasons.

Diseases	Spring				Summer				Autumn				Winter			
	n	OR(95%CI)	Waldχ2	P	n	OR(95%CI)	Waldχ2	P	n	OR(95%CI)	Waldχ2	P	n	OR(95%CI)	Waldχ2	P
Abortion	42	**1.25(1.10, 1.43)**	**10.971**	**0.001**	53	**1.20(1.08, 1.33)**	**11.841**	**0.001**	23	**1.18(1.00, 1.39)**	**3.854**	**0.049**	14	1.11(0.93, 1.33)	1.266	0.261
Acyesis	79	**1.24(1.06, 1.45)**	**6.918**	**0.009**	79	**1.36(1.22, 1.51)**	**31.939**	** < 0.001**	49	**1.35(1.09, 1.67)**	**7.544**	**0.006**	47	1.15(0.90, 1.47)	1.202	0.273
Osteoporosis	117	**1.15(1.05, 1.25)**	**9.005**	**0.003**	114	**1.26(1.16, 1.37)**	**32.002**	** < 0.001**	113	**1.36(1.23, 1.49)**	**38.494**	** < 0.001**	93	**1.15(1.01, 1.30)**	**4.635**	**0.031**
Arthritis	292	**1.09(1.01, 1.17)**	**5.062**	**0.024**	363	**1.25(1.18, 1.32)**	**61.504**	** < 0.001**	195	**1.28(1.18, 1.38)**	**37.079**	** < 0.001**	158	**1.18(1.06, 1.31)**	**8.802**	**0.003**
Disk herniation	75	**1.15(1.04, 1.27)**	**7.524**	**0.006**	83	**1.14(1.03, 1.26)**	**5.930**	**0.015**	39	1.15(0.97, 1.36)	2.651	0.103	44	**1.22(1.06, 1.41)**	**7.590**	**0.006**
Muscle pain	38	**1.17(1.03, 1.33)**	**5.952**	**0.015**	64	**1.12(1.00, 1.26)**	**3.911**	**0.048**	32	**1.37(1.17, 1.61)**	**15.383**	** < 0.001**	29	1.09(0.89, 1.32)	0.695	0.404
Metabolic syndrome	47	**1.15(1.01, 1.32)**	**4.116**	**0.040**	18	**1.42(1.24, 1.63)**	**24.464**	** < 0.001**	25	1.16(0.93, 1.44)	1.731	0.188	19	**1.24(1.01, 1.51)**	**4.474**	**0.023**
Menopausal syndrome	39	**1.15(1.00, 1.32)**	**4.083**	**0.043**	35	**1.17(1.00, 1.37)**	**4.028**	**0.045**	25	1.15(0.92, 1.43)	1.535	0.215	23	**1.33(1.12, 1.58)**	**10.142**	**0.001**
Diabetes mellitus	220	**1.18(1.10, 1.27)**	**19.173**	** < 0.001**	94	1.05(0.94, 1.16)	0.770	0.380	20	**1.25(1.01, 1.55)**	**4.031**	**0.045**	45	**1.32(1.16, 1.51)**	**17.676**	** < 0.001**
Sjogren syndrome	28	1.13(0.96, 1.33)	2.137	0.144	43	**1.16(1.01, 1.33)**	**4.351**	**0.037**	28	**1.30(1.08, 1.56)**	**7.535**	**0.006**	29	**1.19(1.00, 1.41)**	**3.976**	**0.046**

The multinomial logistic regression was used. All the OR values has been adjusted by gender, age, and season. In the 10 specific diseases, %C3-epi-25(OH)D3 can predict the disease risk throughout or nearly throughout the whole four seasons. Its predictive ability can vary from season to season. Significant values are in bold.

## Discussion

In several developed countries, C3-epi-25(OH)D has been adopted as the designated biomarker in nationally representative surveys to investigate its potential clinical significance, such as the National Health and Nutrition Examination Survey^16,17^. Limited evidence regarding the association between C3-epi-25(OH)D and diseases has been reported, with unclear or controversial findings. The lack of standardized measurement methods and reference materials for C3-epi-25(OH)D contributes to significant variations in measured results across different laboratories^25^. In this study, we have developed a highly sensitive method for detecting C3-epi-25(OH)D. The present survey focused on various diseases in adults for the first time and proposed that the percentage of C3-epi-25(OH)D3 may be more suitable for revealing the pathological increase of C3-epi-25(OH)D3 generation under disease conditions. Furthermore, we have substantiated that the percentage, rather than the level, of C3-epi-25(OH)D3 could serve as a potential biomarker to investigate its association with multiple diseases. However, it is imperative to consider the influences of gender, age, and season in order to accurately interpret any findings regarding the relationship between C3-epi-25(OH)D3 percentage and disease risk.

The observed gender, age, and seasonal variations in 25(OH)D3 levels are also evident in C3-epi-25(OH)D3 levels. However, only the seasonal difference is significant, while age and gender differences are only apparent during summer. Therefore, if investigating the relationship between C3-epi-25(OH)D3 levels and diseases, a detailed and intricate experimental design is necessary to control for the influence of gender, age, and seasonal disparities. In comparison, utilizing %C3-epi-25(OH)D3 is relatively straightforward as it primarily requires consideration of the seasonal factor in most scenarios.

The level of C3-epi-25(OH)D3 exhibited a sequential change from high to low during summer, autumn, spring, and winter. This phenomenon can primarily be attributed to the increased production of 25(OH)D3 in response to sunlight exposure^26,27^, resulting in an elevated C3-epi-25(OH)D3 level due to its close association with 25(OH)D3 levels^21^. However, it remains unclear why the %C3-epi-25(OH)D3 value was higher in winter compared to autumn and spring. Similar observations have been reported by other studies as well^28^. In contrast to some reports^28,29^, we did not observe any age or gender differences in %C3-epi-25(OH)D3 among healthy controls. This discrepancy may be attributed to various factors such as ethnic, regional and skin tone variations, interlaboratory differences, and different habits (eg. sunscreen usage)^30,31^. However, during summer, we still observed variations in %C3-epi-25(OH)D3 levels based on age and gender, with higher levels found in males and older individuals. This discrepancy may be attributed to women's relatively greater adherence to sun protection measures (such as using sunshades and sunscreen), while men and older individuals engage in more outdoor activities during summer.

This survey encompassed a total of 11 disease categories and 32 specific diseases, which represent the most prevalent VitD deficiency-associated diseases in adults. In most instances across both disease categories and specific diseases, we observed an inverse correlation between C3-epi-25(OH)D3 levels and C3-epi-25(OH)D3 percentages. The findings confirm that the percentage of C3-epi-25(OH)D3 can serve as an indicator for increased generation of C3-epi-25(OH)D3 under pathological conditions. While the decrease in C3-epi-25(OH)D3 levels during this period is attributed to a close correlation with 25(OH)D3 levels. In contrast, the percentage of C3-epi-25(OH)D3 is the suitable indicator cacable of identifying the pathological increase in 25(OH)D3 epimerization.

An investigation concerning in single nucleotide polymorphisms of C3-epi-25(OH)D has confirmed that the genetic determinants and potential factors of C3-epimers differ from non-C3-epimers^32^. A recent study has further validated that both 25(OH)D and 1α,25(OH)2D are deactivated by 1,25-dihydroxyvitamin D3 24-hydroxylase (an enzyme encoded by the CYP24A1 gene) and 25(OH)D3 C3-epimerase^10^. Due to the intricate nature of circulating C3-epi-25(OH)D levels in terms of specificity, properties, and influencing factors, its contribution in health management and disease occurrence remains unclear. Continuous exploration is needed to develop more reliable methods for utilizing C3-epi-25(OH)D as an assessment tool for health conditions.

To the best of our knowledge, this study represents the first attempt to comprehensively investigate the association between C3-epi-25(OH)D3 and a wide range of common diseases in adults. Our findings highlight that %C3-epi-25(OH)D3, rather than C3-epi-25(OH)D3 level, may serve as a potential biomarker for better understanding the association between C3-epi-25(OH)D3 generation and multiple diseases.

Prior to our report, several studies have investigated the relationship between C3-epi-25(OH)D and specific diseases. However, limited literature exists regarding the pathological significance of C3-epi-25(OH)D (such as in DM complications^33^), with most literature focusing on its role in overestimating Vit D storage^34,35^. Our investigation has confirmed that %C3-epi-25(OH)D3 is significantly associated with 29 vitamin D deficiency-related diseases in adults, both before and after adjusting for gender, age, and season. However, considering the age and gender variations of %C3-epi-25(OH)D3 during summer, it is recommended that future studies on its relationship with multiple diseases should include a stratification analysis based on seasons. In addition, when we further adjusted for 25(OH)D3 levels, only 15 diseases remained significantly associated with %C3-epi-25(OH)D3. Therefore, it seems inappropriate to use the level of 25(OH)D3 as a confounding factor when exploring the relationship between C3-epi-25(OH)D3 generation and disease, because %C3-epi-25(OH)D already encompasses the factor, 25(OH)D levels.

The limitations of this study are as follows. Firstly, this study is only a preliminary and crude analysis concerning in multiple adult diseases. Further research is needed for each specific disease. Secondly, because of so many kinds of diseases, it is impossible to match the age and gender of patients with each disease to healthy controls, but our results showed that %C3-epi-25(OH)D3 level had no significant age and gender difference in adults, except for during summer. Thirdly, this study was based on a single-center cross-sectional study, as such cannot elucidate the causal associations, racial differences, and regional differences. In addition, other potentially influential factors were not considered, such as diet habits, and sunscreen use.

## Conclusions

In conclusion, %C3-epi-25(OH)D3 may more accurately reflect both physiological generation and pathological increase of 25(OH)D3 C3-epimerization. This approach not only corrects for variations highly correlated with 25(OH)D3 levels but also mitigates potential confounding effects of gender and age in most cases. However, further research should be conducted to better understand the pathological significance of %C3-epi-25(OH)D3, with particular attention given to accounting for seasonal influences during experimental design.

### Supplementary Information

Supplementary Information 1.

Supplementary Information 2.

## Data Availability

The datasets used and analyzed during the current study are available from the corresponding author on reasonable request.

## Abbreviations

25(OH)D: 25-Hydroxyvitamin D
BAVD: Bioavailable vitamin D
C3-epi-25(OH)D: C3-epimer of 25-hydroxyvitamin D
EnS: Endocrine society
HPLC–MS/MS: High-performance liquid chromatography-tandem mass spectrometry
IOM: Institute of medicine
JES: Japan endocrine society
NHANES: National health and nutrition examination survey
VitD: Vitamin D
