# Supplementary figures and images for: C3-epi-25(OH)D3 percentage, not level, may be a potential biomarker to reflect its pathological increase in multiple diseases: a cross-sectional case–control study
Source: Sci Rep. 2023 Dec 27;13:23004. doi: 10.1038/s41598-023-50524-3 (PMC10754939; doi:10.1038/s41598-023-50524-3)

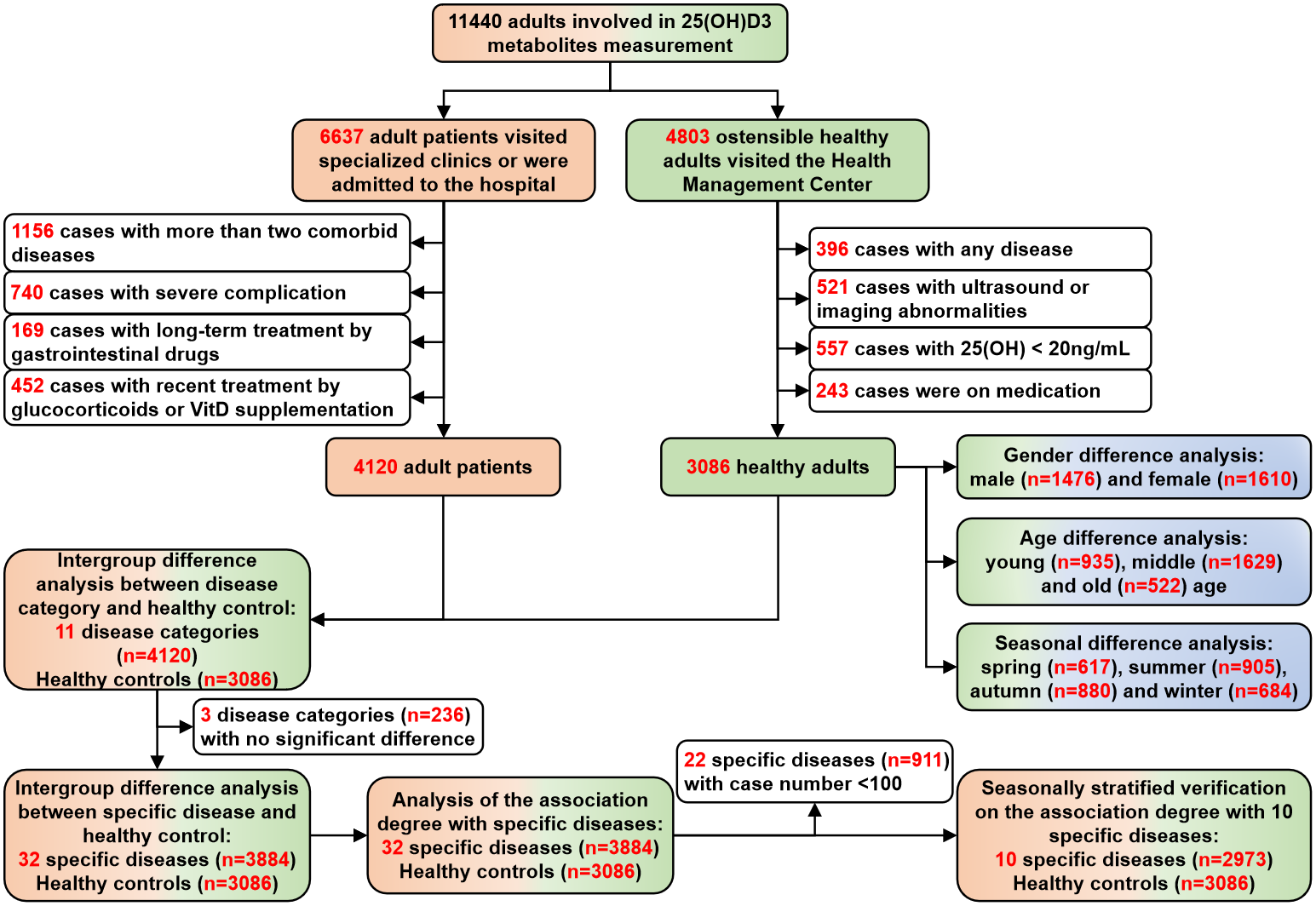

Supplement: Supplementary file 1 — Supplementary Information 1. [file 41598_2023_50524_MOESM1_ESM.tif]

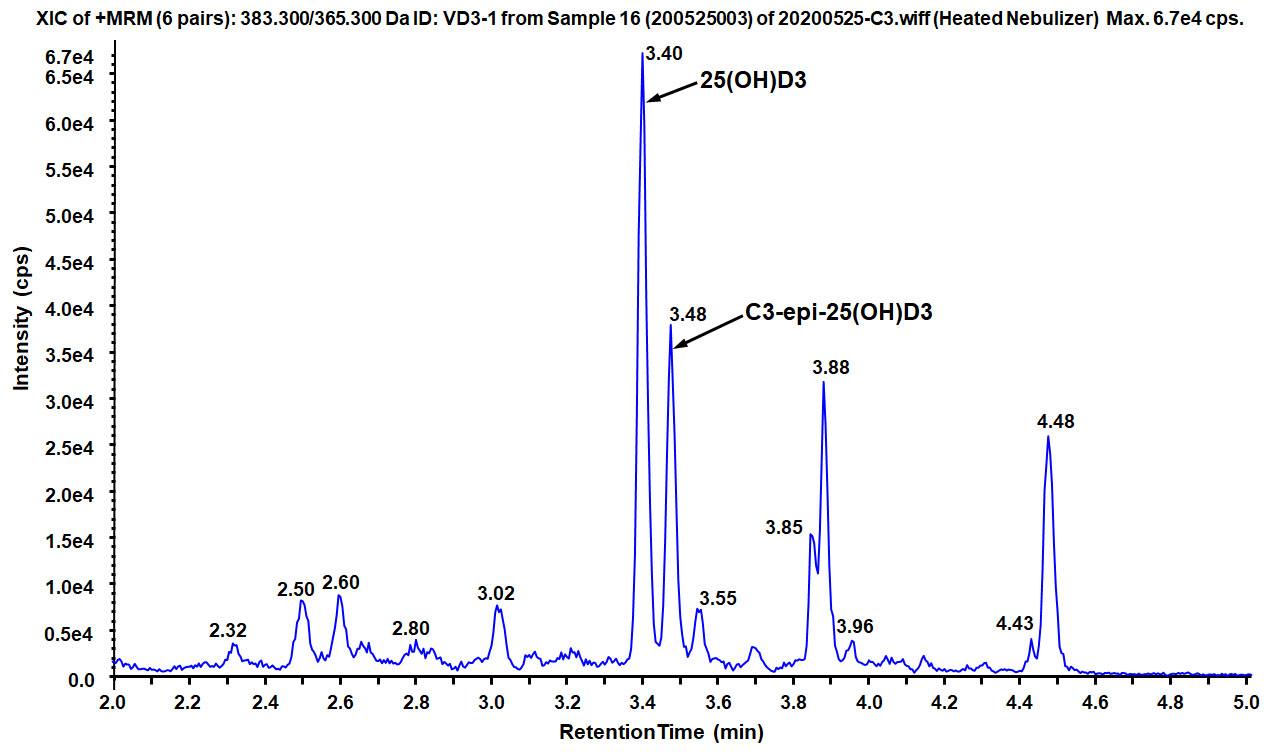

Supplement: Supplementary file 2 — Supplementary Information 2. [file 41598_2023_50524_MOESM2_ESM.jpg]
